# Mesenchymal-epithelial signalling in tumour microenvironment: role of high-mobility group Box 1

**DOI:** 10.1007/s00441-016-2389-7

**Published:** 2016-03-16

**Authors:** Sikander Sharma, Andrew Evans, Elaine Hemers

**Affiliations:** Biomolecular Sciences, School of Pharmacy and Bimolecular Sciences, Liverpool John Moores University, Liverpool, L3 3AF UK

**Keywords:** HMGB1, Myofibroblasts, Invasion, Glucose deprivation, Tumour microenvironment

## Abstract

Glucose deprivation, hypoxia and acidosis are characteristic features of the central core of most solid tumours. Myofibroblasts are stromal cells present in many such solid tumours, including those of the colon, and are known to be involved in all stages of tumour progression. HMGB1 is a nuclear protein with an important role in nucleosome stabilisation and gene transcription; it is also released from immune cells and is involved in the inflammatory process. We report that the microenvironmental condition of glucose deprivation is responsible for the active release of HMGB1 from various types of cancer cell lines (HT-29, MCF-7 and A549) under normoxic conditions. Recombinant HMGB1 (10 ng/ml) triggered proliferation in myofibroblast cells via activation of PI3K and MEK1/2. Conditioned medium collected from glucose-deprived HT-29 colon cancer cells stimulated the migration and invasion of colonic myofibroblasts, and these processes were significantly inhibited by immunoneutralising antibodies to HMGB1, RAGE and TLR4, together with specific inhibitors of PI3K and MEK1/2. Our data suggest that HMGB1 released from cancer cells under glucose deprivation is involved in stimulating colonic myofibroblast migration and invasion and that this occurs through the activation of RAGE and TLR4, resulting in the activation of the MAPK and PI3K signalling pathways. Thus, HMGB1 might be released by cancer cells in areas of low glucose in solid tumours with the resulting activation of myofibroblasts and is a potential therapeutic target to inhibit solid tumour growth.

## Introduction

A dynamic stroma that changes with alterations occurring in the epithelium is fundamental for the maintenance of epithelial tissue. In cancer, these changes maintain an environment that supports proliferation and metastasis (Mareel and Leroy 2003; Tang et al. 2013). Under normal conditions, fibroblasts exhibit little extracellular matrix (ECM) or cell matrix contacts. However, in the event of tissue injury or inflammation, fibroblasts transdifferentiate into myofibroblasts, migrate to damaged tissue and help in the formation of the ECM by producing collagen, fibronectin, tenascin and versican (Vaughan et al. 2000; Hinz et al. 2007). Myofibroblasts are spindle-shaped cells transiently found in early to mid-phase wound tissue (Werner et al. 2007). In addition, they are present in abundance in most solid tumours (Downs-Kelly et al. 2013; Nagarajan et al. 2013; Yeung et al. 2013; Yoshida et al. 2013). Myofibroblasts have also been shown to promote invasiveness in breast carcinoma and pancreatic carcinoma (Dabiri et al. 2013; Hu et al. 2008; Mareel et al. 2009). In the tumour microenvironment, cancer cells stimulate stromal cells, such as myofibroblasts, to secrete growth factors including insulin-like growth factor-2 (IGF-2) and hepatocyte growth factor (HGF; Duckworth et al. 2013). Moreover, these myofibroblasts produce proteases, including various matrix metalloproteinases, which can cleave matrix components (Chien et al. 2013; Guan et al. 2015; Holmberg et al. 2013; Luca et al. 1997). Whereas myofibroblasts appear to play important roles within the tumour microenvironment, the factors that regulate them remain largely unknown. In view of the evidence that the myofibroblast population increases at various progressive stages of invasive carcinomas (De Wever et al. 2008), cancer cells can be hypothesised to release factors that influence myofibroblast proliferation, migration and invasion.

High mobility group box1 (HMGB1) protein is a non-histone protein involved in the stabilisation of nucleosomes and the bending of DNA to facilitate gene transcription (Lange et al. 2008; G. Li et al. 2013). It is expressed in all vertebrate cells and in yeast, plants and bacteria (Bustin et al. 1990). It does not possess a signal sequence and therefore does not transverse the endoplasmic reticulum. However, it is released actively by various cells, such as macrophages (Gardella et al. 2002), pituicytes and erythroleukemia cells, and mediates innate and adaptive inflammatory responses to injury and inflammation (Tang et al. 2007). Although HMGB1 is a nuclear protein, it has also been reported to take part in the immune system when passively released by necrotic cells or actively secreted by inflammatory cells such as dendritic cells in the extracellular milieu (Dumitriu et al. 2005; Tang et al. 2007). Recently, the effect of HMGB1 on fibroblasts and keratinocytes has been studied and HMGB1 appears to stimulate keratinocyte scratch wound healing in vitro. In addition, HMGB1 has been shown to induce proliferation and migration of keratinocytes via the extracellular signal-regulated kinase (ERK) pathway (Hamada et al. 2008; Ranzato et al. 2009; Yang et al. 2007).

HMGB1 has been reported to modulate this cytokine-like activity by interacting with multiple receptors including the receptor for advanced glycation end products (RAGE; Kokkola et al. 2005) and the toll-like receptor 2/4 (TLR2 and TLR4; Curtin et al. 2009; Kim et al. 2013). RAGE belongs to the immunoglobulin superfamily and is expressed in many cells including monocytes, macrophages, smooth muscle cells and endothelial cells. Fibroblasts have been shown to express RAGE and the activation of RAGE has been correlated with the proliferation and migration of fibroblasts in the tumour microenvironment (Liu et al. 2010; Rojas et al. 2010).

In this study, we have hypothesised that HMGB1 is released from cancer cells under tumour microenvironmental stress conditions and that this protein promotes proliferation, migration and invasion of myofibroblasts. Our work shows that the microenvironmental condition of glucose deprivation is responsible for the active release of HMGB1 from HT-29 colon cancer cells and other cancer cell lines. In addition, we demonstrate that HMGB1 stimulates myofibroblast proliferation, migration and invasion through a mechanism that involves the activation of RAGE and TLR4 receptors and the mitogen-activated protein kinase (MAPK) and phosphatidylinositol 3-kinase (PI3K) cell signalling pathways.

## Materials and methods

### Cells and drugs

Human colonic myofibroblast cells CCD18COCo (ATCC CRL1459), colon adenocarcinoma cells HT-29 (ATCC HTB-38), lung cancer cells A549 (ATCC CCL185) and breast cancer cells MCF7 (ATCC HTB22) were obtained from ATCC (LGC Standards, Middlesex, UK) and bladder cancer cells EJ138 were obtained from ECACC (Public Health England, Salisbury, UK). All cells were used within 6 months of resuscitation and were authenticated by the ATCC or ECACC by using standard protocols prior to purchase. Cells were cultured in minimum essential medium from Sigma-Aldrich (Dorset, UK) supplemented with 10 % fetal bovine serum (Labtech International, East Sussex, UK). Human colonic myofibroblast cells were used up to passage 10. The human recombinant HMGB1, anti-HMGB1 primary antibody, anti-RAGE primary antibody and anti-TLR4 primary antibody were purchased from R&D Systems (Oxford, UK). MEK1/2 inhibitor (U0126) and PI3K inhibitor (LY294002) were purchased from Cell Signaling Technology (Massachusetts, USA). Protease and phosphatase cocktail set inhibitors were purchased from Calbiochem (Nottingham, UK). All other reagents were obtained from Fisher Scientific (Leicestershire, UK), BioRad Laboratories (Hertfordshire, UK) or Sigma-Aldrich.

### SDS-polyacrylamide gel electrophoresis and western blot

Conditioned serum-free medium (without glucose or without glutamine) was collected from HT-29, MCF-7, EJ138 and A549 cells after 48 h of conditioning. In addition, cell lysates for analysis were prepared from cells by using RIPA lysis buffer (Life Technologies, Paisley, UK) containing protease inhibitor cocktail set III (10 μl/ml) and phosphatase inhibitor cocktail set II (10 μl/ml).

The CCD18Co cells were treated with either fresh serum- and glucose-free medium or fresh serum-free glucose-containing medium for 24 h before analysis. In addition, the CCD18Co cells were treated (22 h) with serum and glucose-free medium or serum-free glucose-containing medium that had previously been conditioned on HT-29 cells for 48 h. Medium samples and cell lysates were resolved by using SDS-polyacrylamide (9 %) gel electrophoresis and transferred to high-bond nitrocellulose membrane (GE Healthcare, Buckinghamshire, UK). The blots were incubated overnight at 4 °C with primary antibodies to HMGB1, RAGE or TLR4, followed by a 1-h incubation at room temperature with the appropriate horseradish-peroxidise-conjugated secondary antibody. The blots were then incubated with SuperSignal West Pico Chemiluminescent Substrate with enhanced chemiluminescence (ECL; Thermo Scientific, Northumberland, UK), developed and fixed in the dark room.

### Proliferation assays

CCD18Co cells were cultured in 24-well plates (5×10^4^/well) and serum-starved for 24 h prior to HMGB1 treatment. They were then treated with recombinant HMGB1 at a range of doses (0.01 to 100 ng/ml) and incubated at 37 °C, 95 % humidity and 5 % CO_2_ for either 48 h or 96 h. After treatment with HMGB1, the proliferative effect of recombinant HMGB1 on CCD18Co cells was assessed by using Neutral Red Uptake (NRU) or MTT (3-(4,5-dimethylthiazol-2-yl)-2,5-diphenyltetrazolium bromide) assays according to standard protocols.

The effect of MEK1/2 inhibitor (U0126) or PI3K inhibitor (LY294002) was also measured by using the MTT assay. The CCD18Co cells (5×10^4^/well) were cultured in 24-well plates in serum-free medium for 24 h followed by treatment with U0126 (50 μM) and LY294002 (10 μM) in serum-free medium for 48 h. In addition, the inhibitory effects of the MEK1/2 inhibitor (U0126) and PI3K inhibitor (LY294002) on the recombinant-HMGB1-induced proliferation of CCD18Co cells were measured by using the MTT assay. The CCD18Co cells (5×10^4^/well) were cultured in 24-well plates in serum-free medium for 24 h prior to treatment with HMGB1 (10 ng/ml) with U0126 (50 μM) or LY294002 (10 μM) in serum-free medium for 48 h before assessment by the MTT assay.

### Migration assays

The migratory effect of CCD18Co cells in response to medium previously conditioned on HT-29 cells for 20 h was studied by using 8-μm-pore Boyden chamber inserts in a 24-well plate system (BD Bioscience, California, USA). CCD18Co cells (6×10^4^/well) were seeded onto the inserts in serum-free medium. Conditioned medium or conditioned medium with the addition of a variety of inhibitors and antibodies (MEK1/2 and PI3K inhibitors and anti-HMGB1, anti-RAGE and anti-TLR4 antibodies) was added to the lower chamber and the plate was incubated for 20 h at 37 °C, 95 % humidity and 5 % CO_2_ to allow the cells to migrate. CCD18Co cell migration across the 8-μm-pore membrane was detected by using REASTAIN Quick-Diff stain (Dade Behring, Delaware, USA). The total cells in five microscopic fields of vision (× 20) per well were photographed and counted and the mean cell count of 6 wells per experiment was determined.

### Invasion assays

Invasion assays with CCD18Co cells were performed by using 8-μm-pore matrigel matrix Biocoat inserts according to the manufacturer’s instructions (Becton Dickinson, Oxford, UK). The CCD18Co cell suspension was prepared in serum-free medium (6×10^4^/ml). The CCD18Co cell suspension was seeded onto the matrigel inserts (0.5 ml per insert) in a 24-well plate supplied by the manufacturer. HT-29-cell-conditioned medium was used as a chemoattractant and was added into the lower chamber. The plate was incubated for 22 h at 37 °C, 95 % humidity and 5 % CO_2_. In addition to the standard invasion assay, the medium in the CCD18Co cell suspension and conditioned medium used at the bottom chamber were supplemented with a variety of inhibitors and neutralising antibodies. The CCD18Co cell invasion, in response to the conditioned medium with and without MEK1/2 and PI3K inhibitors or anti-HMGB1, anti-RAGE and anti-TLR4 antibodies, was determined by their inclusion in the lower chamber of the 24-well plate. Cells invading through the matrigel matrix membrane were detected on the lower surface by using the REASTAIN Quick-Diff staining method and were counted as described above.

## Results

### HMGB1 triggers proliferation in myofibroblasts via MAPK and PI3K signalling pathways

**Fig. 1:**
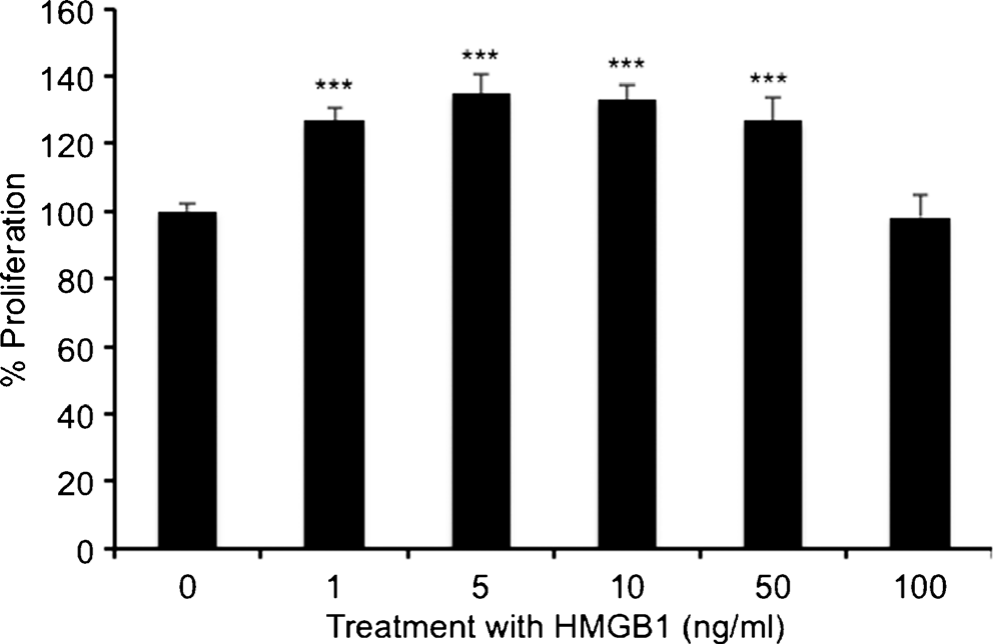
Recombinant high mobility group box1 (*HMGB1*) stimulates proliferation of myofibroblasts at 96 h (1-100 ng/ml). All results of the Neutral Red Uptake assays are expressed as the percentage proliferation compared to the control (0 ng/ml). *Error bars* represent SEM; *n* = 29, ***P < 0.001 compared with the control

**Fig. 2:**
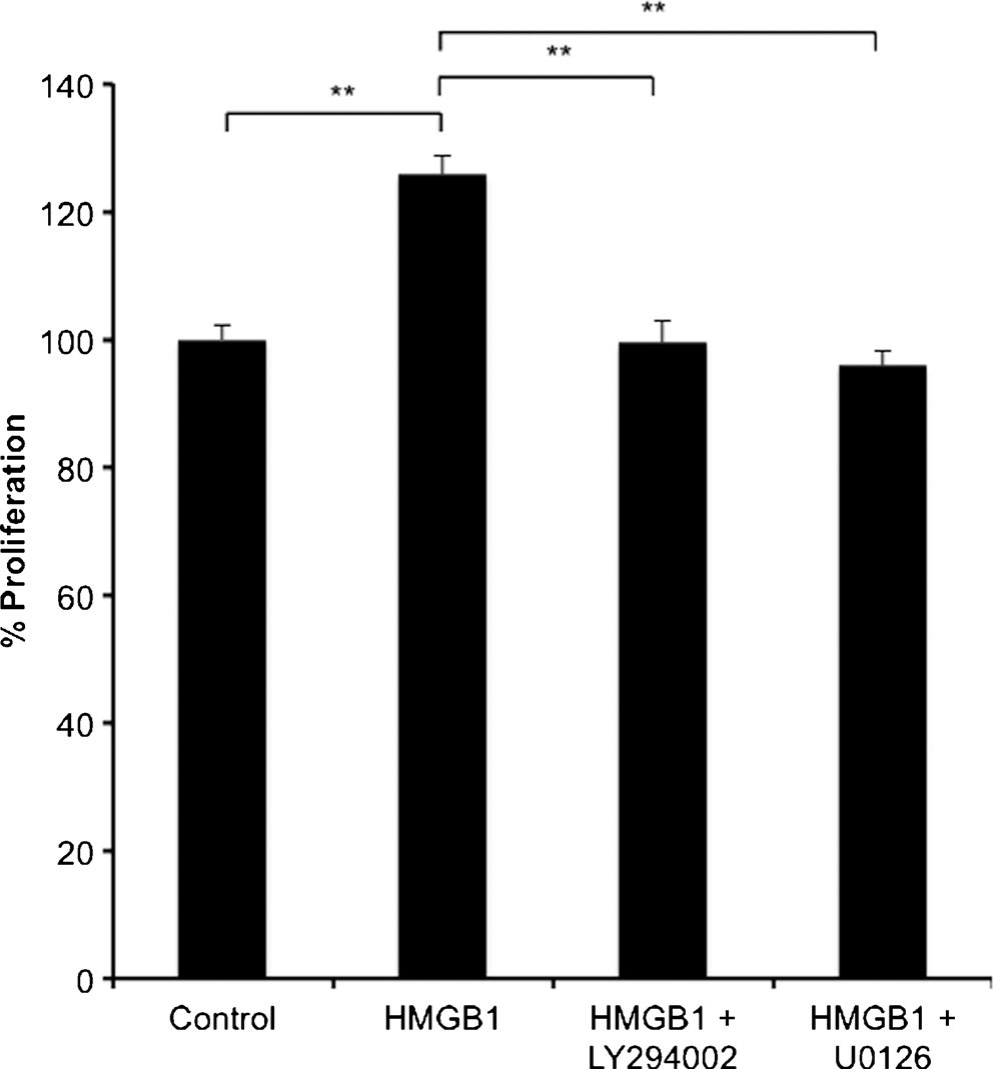
Recombinant HMGB1 stimulated myofibroblast proliferation (48 h) in the presence of mitogen-activated protein kinase (MAPK) and phosphatidylinositol 3-kinase (PI3K) inhibitors. The MEK1/2 inhibitor U0126 (50 μM) and the PI3K inhibitor LY294002 (10 μM) significantly inhibited HMGB1 (10 ng/ml)-stimulated myofibroblast proliferation. *Error bars* represent SEM; *n* = 18, **P < 0.01

To determine the proliferative effect of HMGB1, myofibroblasts were treated with a range of concentrations (1–100 ng/ml) of recombinant HMGB1 for 96 h. We observed that a wide range of concentrations stimulated statistically significant myofibroblast proliferation (1–50 ng/ml) compared with the control (Fig. 1). A statistically significant increase in proliferation of myofibroblasts was also stimulated by HMGB1 (10 ng/ml) at an earlier time point of 48 h (Fig. 2).

The MAPK and PI3K signalling pathways have previously been reported to be involved in colonic myofibroblast proliferation (Hemers et al. 2005). Since 10 ng/ml HMGB1 significantly induced proliferation in myofibroblasts at 48 h, the signalling pathway inhibitory assays were also performed for 48 h. To investigate the role of the PI3K signalling pathway in HMGB1-stimulated myofibroblast proliferation, the PI3K inhibitor LY294002 (50 μmol/l) was used. This inhibitor completely abolished HMGB1-stimulated myofibroblast proliferation (Fig. 2). The results from the MTT proliferation assays suggested that LY294002 at 50 μM was non-toxic to myofibroblasts at 48 h (data not shown). Therefore, these data indicated that HMGB1-stimulated myofibroblast proliferation was also mediated through activation of the PI3K signalling pathway.

To investigate the role of the MAPK signalling pathway in HMGB1-stimulated myofibroblast migration, the inhibitor of p42/44 kinase, MAPK kinase (U0126, 10 μmol/l) was used. This inhibitor completely inhibited HMGB1-stimulated myofibroblast proliferation (Fig. 2). The results from the MTT proliferation assays suggested that U0126 at 10 μM was non-toxic to myofibroblasts at 48 h (data not shown). Therefore, these data indicated that HMGB1-stimulated myofibroblast proliferation was mediated through the activation of the MAPK signalling pathway.

### Glucose deprivation triggers release of HMGB1 from cancer cells

**Fig. 3:**
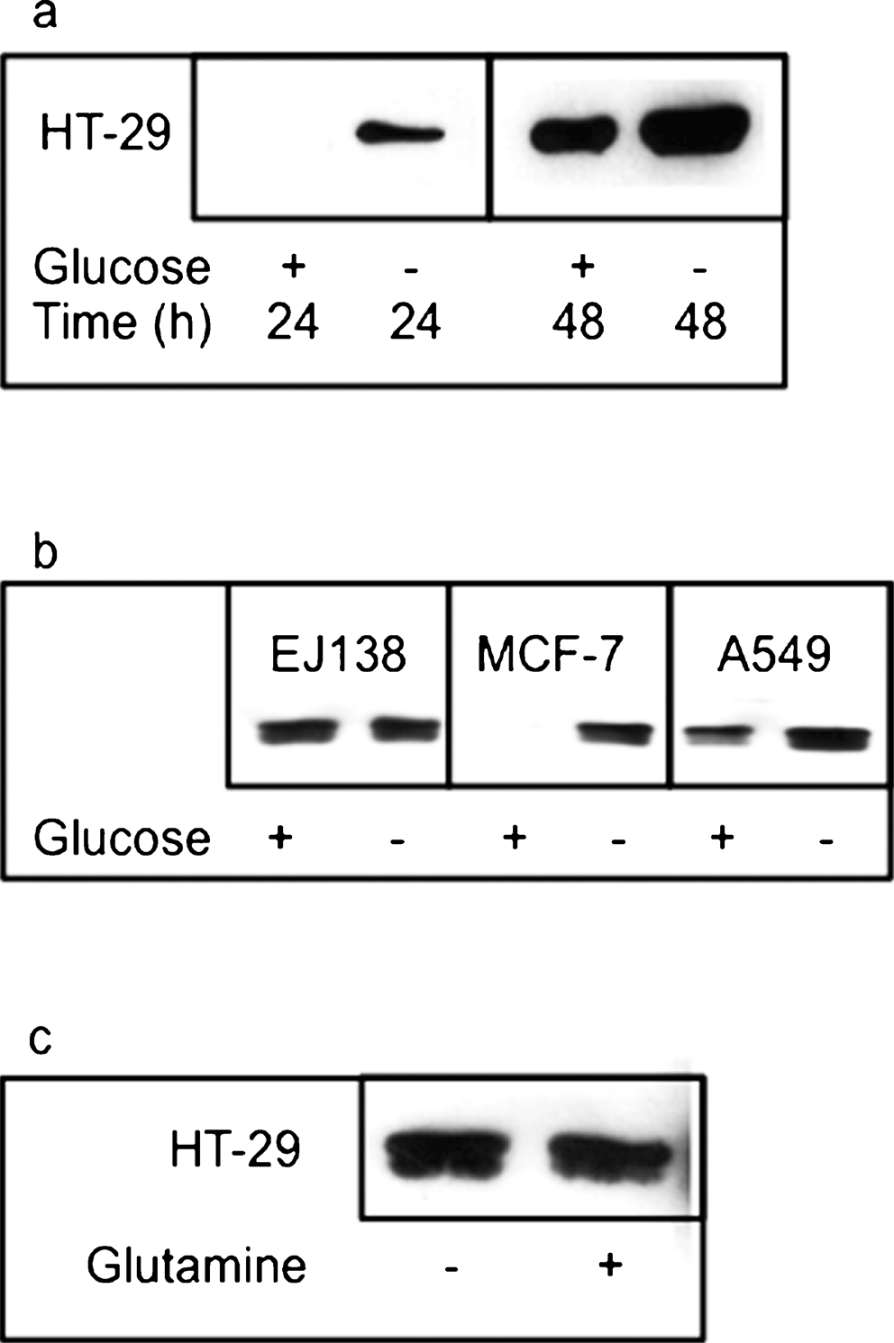
Western blot analysis of conditioned medium demonstrated an increase in HMGB1 in (**a**) HT-29 cells cultured in medium without glucose for 24 h and 48 h compared with medium containing glucose and in (**b**) MCF-7 and A549 cells cultured in medium without glucose for 48 h compared with medium containing glucose. However, no change was seen in the conditioned medium taken from EJ138 cells cultured in the same way. **c** No change in the amount of HMGB1 was detected in medium taken from HT-29 cells that had been cultured in medium from which glutamine was absent compared with medium that contained glutamine at 48 h. All cells were exposed to normoxic conditions. Each western blot was repeated a minimum of three times on three separate occasions

The release of HMGB1 under normoxic and anoxic conditions and also in the absence or presence of glucose was compared. Surprisingly, the amount of HMGB1 released from cancer cells under normoxic conditions without glucose was higher than those values under anoxic conditions with or without glucose (anoxic data not shown). The increased release of HMGB1 under low glucose conditions was detected in the medium from HT-29 cells at both 24 h and 48 h in the presence of oxygen in HT-29 cells (Fig. 3a). In addition, we found that this increase in HMGB1 release in the absence of glucose did not occur under conditions in which the cells were deprived of glutamine (Fig. 3c). This further confirmed that glucose deprivation in the presence of oxygen was a strong stimulant for HT-29 colon cancer cells to release HMGB1.

### Release of HMGB1 in response to glucose deprivation is not cancer-cell-line-specific

The results from western blot data showed that glucose deprivation triggered the release of HMGB1 from HT-29 colon cancer cells (Fig. 3a). To investigate whether this HMGB1 release was cell-line-specific or a phenomenon shared by other cell lines, MCF-7 (breast cancer), EJ138 (bladder cancer) and A549 (lung cancer) cells were also subjected to glucose deprivation. The culture medium was again collected after 48 h and any HMGB1 released into the medium was detected by western blot analysis (Fig. 3b). The results suggested that, like the response of the HT-29 colon cancer cell line, glucose deprivation also triggered the release of HMGB1 from MCF-7 and A549 cells. However, there appeared to be no difference in the level of HMGB1 released from EJ138 cells cultured with or without glucose. The MCF-7 and A549 cells followed the same pattern with an increased amount of HMGB1 released under glucose-deprived conditions compared with normal levels of glucose. The MCF-7 cells showed the greatest difference in the quantity of HMGB1 released, showing no detectable band in the glucose-containing medium and a strong band in medium deprived of glucose after 48 h (Fig. 3b). Therefore, three out of the four cell lines investigated appeared to follow the same pattern, releasing relatively higher amounts of HMGB1 after glucose deprivation under normoxic conditions (Fig. 3a, b).

### Glucose-free medium conditioned by HT-29 colon adenocarcinoma cells triggers migration and invasion of myofibroblasts

**Fig. 4:**
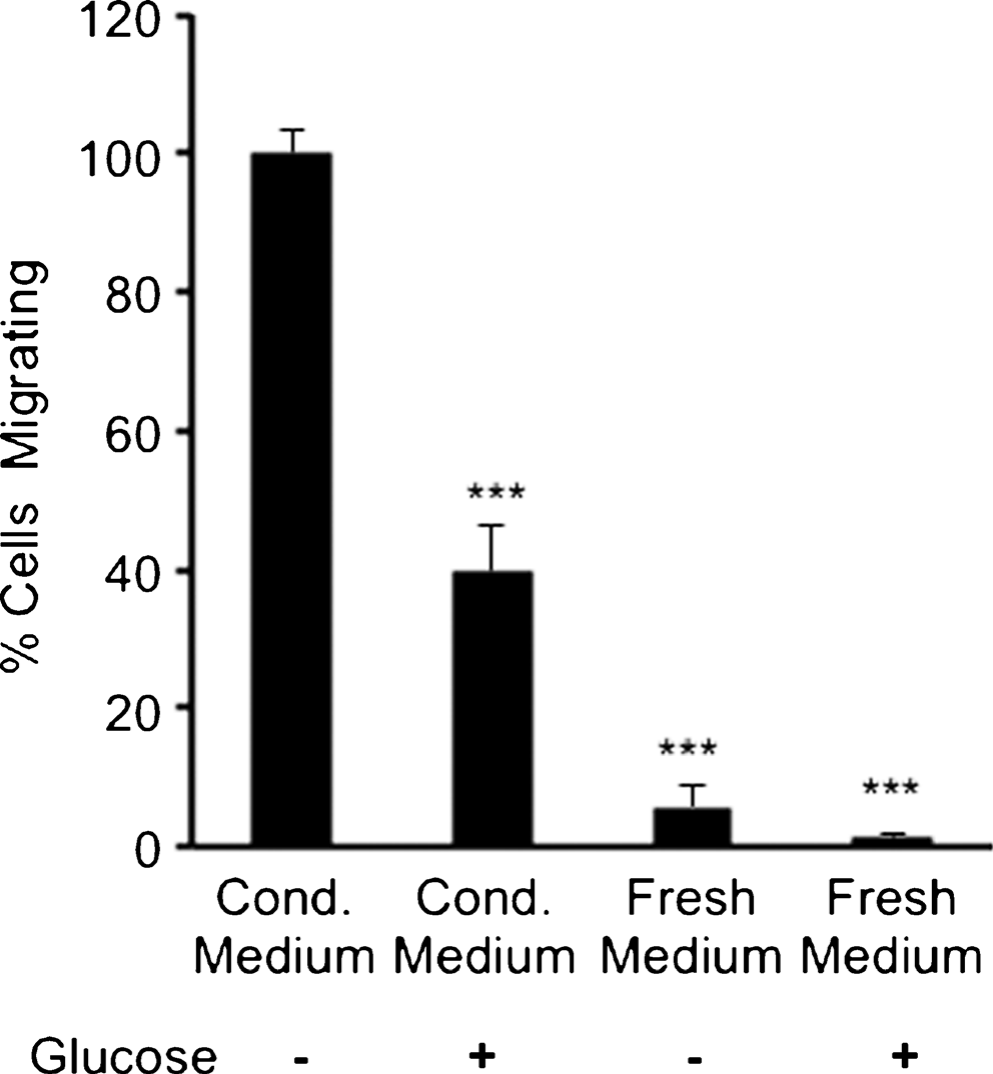
Conditioned medium (*Cond. medium*) from HT-29 cells cultured in medium without glucose stimulated migration of myofibroblasts. The migration assay was carried out with Boyden chamber inserts and the cells were stimulated for 20 h. The myofibroblasts were exposed to conditioned medium from HT-29 cells that either contained glucose or lacked glucose and to fresh medium that also either contained glucose or lacked glucose. All conditions were compared with conditioned medium lacking glucose, which was expressed as 100 %. *Error bars* represent SEM; ***P < 0.001; *n* = 3 for all conditions, except for conditioned medium minus glucose where *n* = 7

**Fig. 5:**
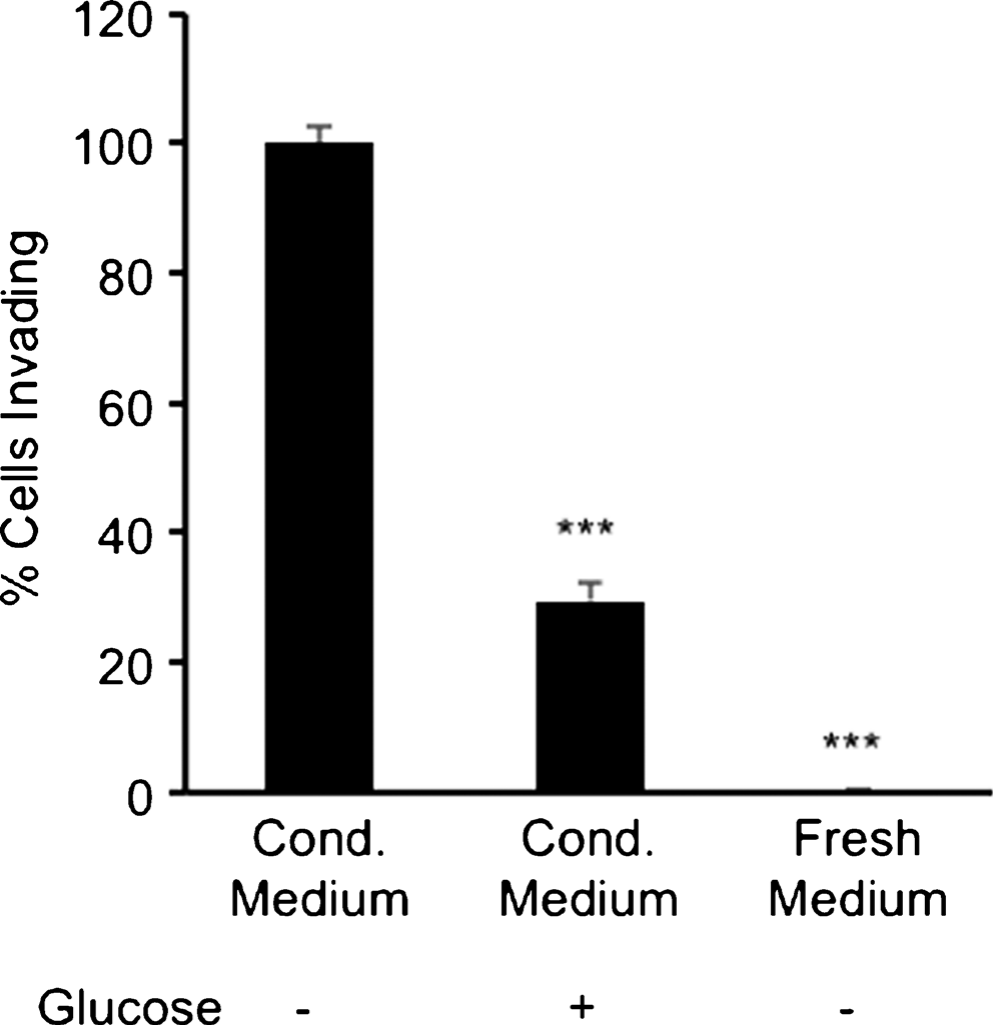
Conditioned medium (*Cond. medium*) from HT-29 cells cultured in medium without glucose stimulated invasion of myofibroblasts into a Matrigel matrix. The myofibroblasts were exposed to conditioned medium from HT-29 cells that either contained glucose or lacked glucose and to fresh medium that did not contain glucose for 22 h. All conditions were compared with conditioned medium lacking glucose, which was expressed as 100 %. *Error bars* represent SEM; ***P < 0.001, *n* = 5

Migration and invasion assays were performed by using glucose-free conditioned medium collected from HT-29 colon cancer cells as a potential chemoattractant. This conditioned medium served as a positive control (100 % migration or 100 % invasion) and was compared with conditioned medium with glucose and fresh medium with or without glucose. The results obtained from these assays suggested that HT-29 cells undergoing glucose starvation released chemoattractants that stimulated the migration and invasion of myofibroblasts (Figs. 4, 5). Glucose-containing conditioned medium also stimulated the migration and invasion of myofibroblasts compared with fresh medium but this was significantly lower than that caused by glucose-free conditioned medium (Figs. 4, 5). These data suggested that conditioned medium from HT-29 cells, especially glucose-depleted conditioned medium, significantly stimulated the migration and invasion of myofibroblasts.

### HMGB1 released from HT-29 colon adenocarcinoma cells triggers invasion and migration in myofibroblasts

**Fig. 6:**
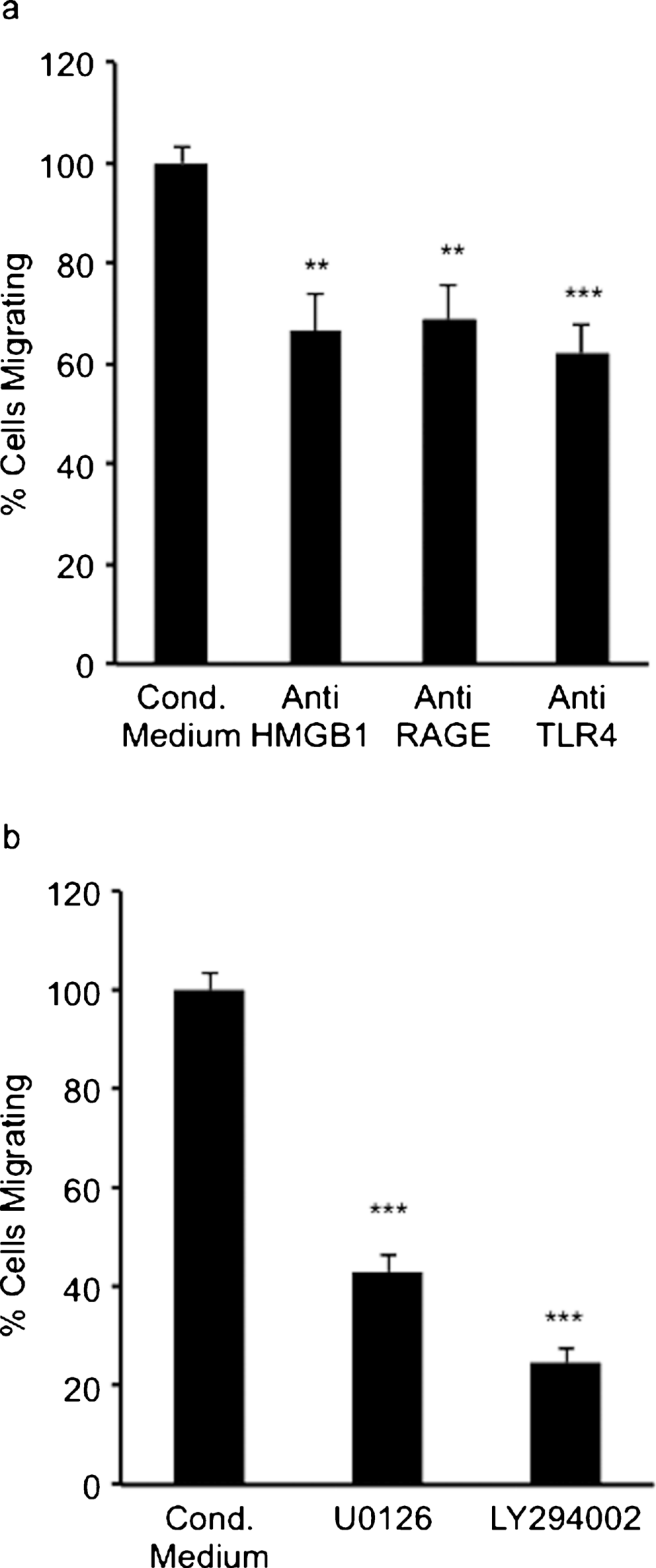
Myofibroblast migration stimulated by glucose-free conditioned medium from HT-29 cells was significantly inhibited by (**a**) immunoneutralising antibodies to high mobility group box1 (*HMGB1*), receptor for advanced glycation end products (*RAGE*) and toll-like receptor 4 (*TLR4*) and (**b**) by the MEK1/2 inhibitor UO126 (50 μmol/l) and the PI3K inhibitor LY294002 (10 μmol/l). *Error bars* represent SEM; **P <0.01, ***P < 0.001, *n* = 3 for all conditions, except for conditioned medium minus glucose where *n* = 7

**Fig. 7:**
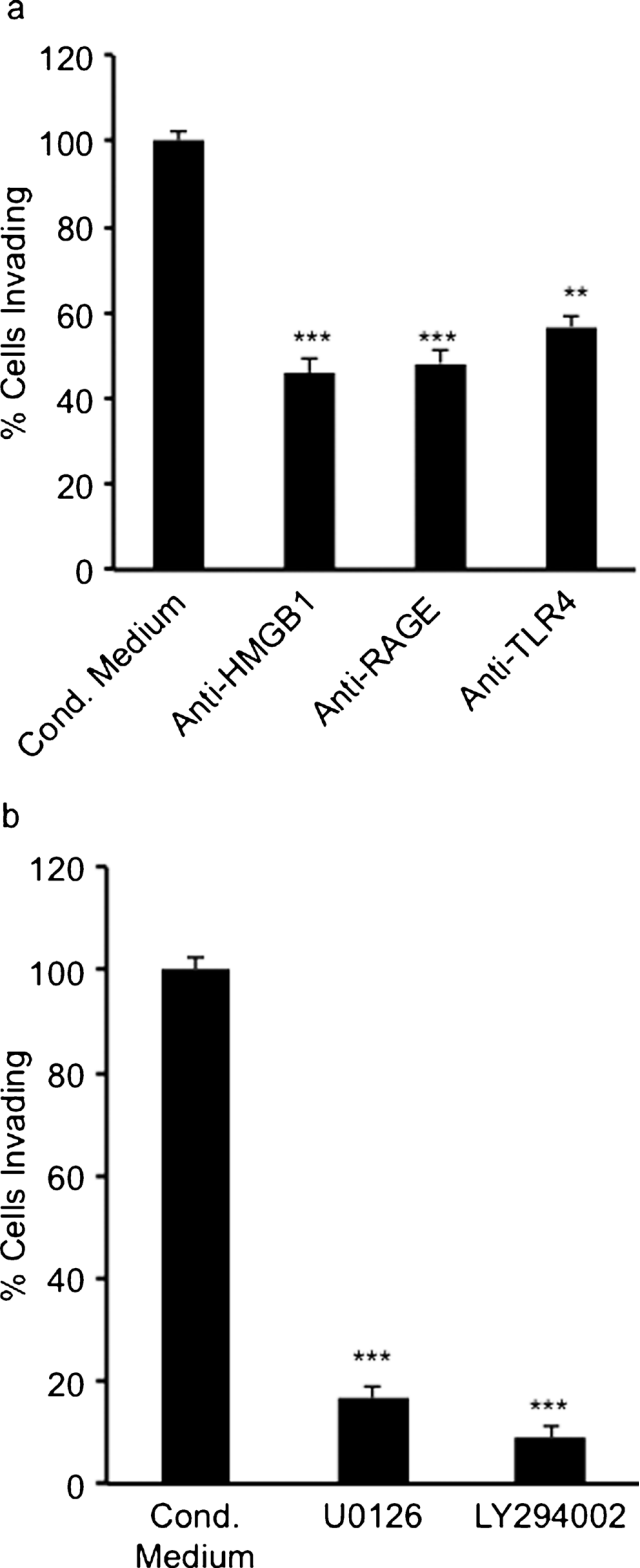
Myofibroblast invasion into a Matrigel matrix stimulated by glucose-free conditioned medium from HT-29 cells was significantly inhibited by (**a**) immunoneutralising antibodies to HMGB1, RAGE and TLR4 and (**b**) by the MEK1/2 inhibitor UO126 (50 μM) and the PI3K inhibitor LY294002 (10 μM). *Error bars* represent SEM; **P < 0.01, ***P < 0.001, *n* = 5

Increased HMGB1 in the conditioned medium was detected from glucose-deprived HT-29 cell lines (Fig. 3a). To investigate whether the HMGB1 present in this culture medium was stimulating migration and invasion of myofibroblasts (Figs. 4, 5), migration and invasion assays were performed with the addition of immunoneutralising anti-HMGB1 antibodies (5 μg/ml) added to the glucose-free conditioned medium. The addition of the anti-HMGB1 antibodies significantly inhibited myofibroblast migration and invasion stimulated by this glucose-free conditioned medium (Figs. 6a, 7a) suggesting that HMGB1 plays an important role in the invasion and migration of these cells in glucose deprivation.

### HMGB1 binding to RAGE and TLR4 triggers invasion and migration of myofibroblasts

Although RAGE is the main receptor for HMGB1, HMGB1 is known to interact with other receptors including TLR4. Western blot analysis confirmed the presence of TLR4 and RAGE on myofibroblast cells (data not shown). Therefore, HMGB1-mediated migration and invasion of myofibroblasts might involve the activation of an HMGB1-RAGE complex and/or HMGB1-TLR4 complex, resulting in the stimulation of downstream signalling cascades. To investigate the role of these receptors in myofibroblast migration and invasion, myofibroblasts were stimulated with glucose-free conditioned medium from HT-29 cells, with or without the addition of immunoneutralising anti-RAGE antibodies (8 μg/ml) or anti-TLR4 antibodies (2 μg/ml). The presence of either anti-RAGE antibodies or anti-TLR4 antibodies significantly inhibited myofibroblast migration and invasion stimulated by glucose-free HT-29-cell-conditioned medium (Figs. 6a, 7a). These results suggested that the HMGB1 present in the glucose-free conditioned medium from HT-29 cells stimulated myofibroblast migration and invasion through the activation of both RAGE and TLR4.

### HMGB1-triggered migration and invasion in myofibroblasts involve activation of the MAPK and PI3K signalling pathways

To investigate the possible downstream signalling pathways involved in the migration and invasion of myofibroblasts stimulated with glucose-free HT-29-cell-conditioned medium, the potential roles of the MAPK and PI3K signalling pathways were considered. Migration and invasion assays were therefore carried out by using the selective inhibitors of MEK1/2 and PI3K. These inhibitors were added to both the medium in the bottom chamber and to the myofibroblast cell suspension in the inserts. The selective MEK1/2 inhibitor U0126 (50 μM) significantly reduced myofibroblast migration (Fig. 6b) and invasion (Fig. 7b) compared with glucose-free conditioned medium. In addition, LY294002 (10 μM), a selective inhibitor of PI3K, resulted in a significant reduction in myofibroblast migration (Fig. 6b) and invasion (Fig. 7b) compared with glucose-free conditioned medium. These results suggested that both the MAPK and PI3K were involved in the migration and invasion of myofibroblasts stimulated by glucose-free HT-29-cell-conditioned medium.

## Discussion

In many solid tumours, including those of the colon and breast, the tumour stroma comprises a major part of the tumour mass (Peña et al. 2013; Tripathi et al. 2012). Myofibroblasts are the predominant stromal cell type in most carcinomas and have been shown to take part in tumour proliferation by secreting a number of growth factors, including IGF-1, IGF-II and HGF (Hinz et al. 2007; Tripathi et al. 2012). However, the role of cancer cells in the stimulation of myofibroblasts has not been fully explored. The proliferative and migratory properties of HMGB1, a novel non-histone nuclear protein, have been an area of recent interest and HMGB1 has been reported to be involved in the proliferation of fibroblasts from the lung (Li et al. 2015) and in the proliferation and migration of fibroblasts from gingival and periodontal tissue (Chitanuwat et al. 2013), in keratinocytes (Ranzato et al. 2009) and in skin fibrobroblasts (Ranzato et al. 2010). Limited evidence of the role of HMGB1 in the stimulation of myofibroblast migration is available (Lee et al. 2015) but no evidence exists to date regarding the role of HMGB1 in stimulating other activities of myofibroblasts, such as proliferation and invasion. The data presented here show that recombinant HMGB1 at 10 ng/ml significantly stimulates myofibroblast proliferation. Furthermore, our results suggest that HMGB1 is actively released by cancer cells under stressful conditions and that this might regulate the proliferation, migration and invasion of myofibroblasts.

Together with hypoxia, glucose deprivation has been considered to be a major hallmark of the solid tumour microenvironment (Jang and Hill 1997). The results of our work show that the nuclear protein HMGB1 is released from glucose-deprived cancer cells. The presence of HMGB1 in the culture medium of macrophages and monocytes has been reported previously (Tang et al. 2007). Cells undergoing autophagy are thought to release HMGB1 into the microenvironment (Thorburn et al. 2009a). Autophagy has been shown to be induced by nutrient starvation (L. Li et al. 2013). However, whether the deprivation of a particular nutrient is responsible for the release of HMGB1 into the microenvironment during autophagy is unclear. Nevertheless, our western blot data suggest that glucose deprivation triggers the release of HMGB1 from HT-29 cells under normoxic conditions. HMGB1 is also released from HT-29 cells under anoxic conditions (data not shown). Anoxic conditions cause oxidative stress to cells and therefore they might be stimulated to undergo autophagy (Yang et al. 2015). Autophagic cells have previously been shown to selectively release HMGB1 without disrupting the cell membrane via an unknown mechanism (Thorburn et al. 2009b). The amount of HMGB1 released under glucose-deprived normoxic conditions is much higher than other conditions investigated. These include anoxia in addition to glucose deprivation (data not shown), a much harsher microenvironmental condition. This suggests that the release of HMGB1 from HT-29 cells is not the result of autophagy but is potentially induced by a specific microenvironmental stress (glucose deprivation) stimulating active secretion. The release of HMGB1 from glucose-deprived HT-29 cells might be nutrient-specific, as glutamine deprivation does not have the same effect. Additionally, other cell lines have been investigated to determine whether the release of HMGB1 under conditions of glucose deprivation is cell-line-specific. Our western blot results suggest that glucose deprivation in normoxic conditions also stimulates the release of HMGB1 from cancer cell lines MCF-7 (breast) and A549 (lung), but not from EJ138 bladder cancer cells.

The data from the migration and invasion assays indicate that HMGB1 present in low-glucose culture medium taken from HT-29 cells triggers the migration and invasion of myofibroblasts. The involvement of HMGB1 in this migration and invasion has been confirmed by using immunoneutralising antibodies against HMGB1, whereby the presence of anti-HMGB1 antibodies results in a statistically significant reduction in the migration and invasion of myofibroblasts. This suggests a key role of HMGB1 in the regulation of these processes. HMGB1 has been shown to interact with many receptors including TLR4 and RAGE. For example, the interaction of HMGB1 with TLR4 has been implicated in stimulating the proliferation and migration of hepatic stellate cells via the activation of the PI3K pathway (Wang et al. 2013). Moreover, the activity of the HMGB1-RAGE complex has been implicated in the regulation of the migration of dendritic cells and myoblasts (Dumitriu et al. 2007; Riuzzi et al. 2006). Results from western blot analysis have revealed that TLR4 and RAGE are expressed by myofibroblasts (data not shown). In this study, we report that the inhibition of TLR4 or RAGE by immunoneutralising antibodies statistically significantly inhibits the migration and invasion of myofibroblasts. This suggests that an interaction between HMGB1 and either of these receptors mediates the activation of downstream signalling pathways to trigger migration and invasion. HMGB1 might also interact with other receptors, which are as yet to be investigated.

The activation of the PI3K and MAPK pathways in both the proliferation and migration of cancer cells, inflammatory cells and myofibroblasts has been previously reported (Hemers et al. 2005; Kang et al. 2013; Mahajan and Dhawan 2013). In addition, both pathways have been previously shown to be involved in the invasion of hepatocellular carcinoma and prostate cancer cells (Chen et al. 2005). However, an involvement of these pathways in HMGB1-stimulated myofibroblast proliferation, migration and invasion has not previously been reported. To determine the involvement of the signalling pathways in HMGB1-stimulated proliferation, migration and invasion, PI3K and MEK1/2 have been selectively inhibited by using U0126 and LY294002, respectively. The results suggest that HMGB1-induced proliferation, migration and invasion of myofibroblasts involves the activation of the PI3K and MAPK signalling pathways.

The results from this work have shown that HMGB1, a histone-associated nuclear protein normally involved in DNA stabilisation, is actively released from cancer cells in response to low-glucose levels. Cancer cells, under certain microenvironmental stress conditions, such as hypoxia or low glucose, are known to become more aggressive and invasive and also more likely to metastasise (Evans et al. 2008). Our study has shown that HMGB1 interacts with TLR4 and RAGE expressed by myofibroblasts. The TLR4 or RAGE interaction with HMGB1 released from cancer cells engages downstream the PI3K and MAPK pathways to signal the myofibroblasts to proliferate, migrate and invade. The release of HMGB1 from cancer cells under low-glucose conditions and its subsequent interaction with myofibroblasts have not been previously reported. These findings might have profound implications for our understanding of the carcinogenesis process and therefore may reveal novel therapeutic targets for the treatment of cancer.
